# Ending tuberculosis in risk groups in Europe: challenges from travel and population movement

**DOI:** 10.2807/1560-7917.ES.2017.22.12.30489

**Published:** 2017-03-23

**Authors:** Charlotte Jackson, Ibrahim Abubakar

**Affiliations:** 1 UCL Institute for Global Health, London, United Kingdom

**Keywords:** air-borne infections, bacterial infections, tuberculosis, migration, Europe, air travel

As many countries in Europe make progress in tuberculosis (TB) control, TB incidence in Europe is diverse; in low-incidence countries (those with an incidence less than 20 per 100,000 [1]) the TB burden is increasingly borne by specific risk groups, such as migrants from high- to lower-incidence countries, persons with social risk factors such as homelessness and individuals who have been in contact with a TB patient. Strategies to control TB within these risk groups include screening for active disease and sometimes latent infection [2,3], followed by treatment where appropriate. The effectiveness of screening strategies to identify patients needing treatment varies. For example, the yield of active TB among migrants from high- to low-burden countries ranged from 7 to 10,186 per 100,000 people screened, depending on various factors including the TB prevalence in the country of origin [2]. In this World TB Day issue of *Eurosurveillance*, four papers describe the risks of TB infection and disease in two potentially high-risk groups: migrants [4,5] and airline passengers [6,7].

Migrants are considered to be at high risk of TB, for reasons such as the possibility of reactivation of latent infection acquired in their home country, frequent travel to high-incidence areas, and perhaps transmission within migrant communities in the receiving countries [8]. At European Union (EU) level, Hollo et al. report that TB rates are higher, and declining more slowly, in individuals not native to the reporting countries compared with the native population [4]. This highlights important health inequalities and major challenges to the control of TB, while it also illustrates difficulties in combining data from multiple countries. Besides differences in the definition of TB cases of ‘foreign origin’ between countries, several points complicate interpretation of these data. Migrants constitute a heterogeneous group of individuals from multiple countries and with varying risk factor profiles, thus simply being ‘foreign born’ is not necessarily a good proxy for having a high risk of TB infection or disease, and particular risk factor profiles may be more common in some countries than in others, e.g. due to differences in migration patterns. The implications of migration for TB incidence thus depend on detailed patterns of migration [9]. There is therefore a need to further investigate, at country level, risk factors for TB in migrants and to further elucidate detailed migration and travel patterns and develop tailored solutions specific to the epidemic affecting each group [10].

Addressing the needs of a specific group of migrants, namely asylum seekers, Bozorgmehr and colleagues systematically review the yield of upon-entry screening for active TB of asylum seekers entering Germany, a low-incidence country [5]. Like many European countries, Germany sees a higher rate of TB among foreign-born compared with native-born individuals. Pooling results from the six diverse studies included in the review, the authors report that 3.47 cases of active TB were identified per 1,000 asylum seekers screened. However, there was substantial heterogeneity between studies, and the authors highlight the need to understand reasons for this variation. Explanations might include differences in study populations e.g. countries of origin, age distribution, prevalence of co-morbidities, case definitions and diagnostic methods. Furthermore, cost-effectiveness of screening approaches including cost per quality-adjusted life year should inform the selection of screening methods and the prioritisation of populations.

Arguably more controversial than screening migrants is the issue of screening individuals exposed to patients with active TB on board aircraft. While the World Health Organization (WHO) and European Centre for Disease Prevention and Control (ECDC) recommend contact investigations among passengers seated within two rows of an infectious case on flights lasting 8 hours or longer [11,12], both organisations and others [13] acknowledge that there is only limited evidence to quantify the risk of transmission on aircraft. Two papers in this issue discuss screening of airline contacts for latent TB infection (LTBI): one is an intensive contact investigation following a fatal case of extensively drug-resistant (XDR)-TB [7], while the other reports the overall yield based on multiple investigations conducted in Japan [6]. Both report very low yields. The XDR-TB study included tuberculin skin tests (TST) and interferon gamma release assays (IGRAs) within 8 weeks from exposure and at least 8 weeks after exposure. One case of possible transmission (TST conversion) from the XDR-TB patient occurred among 112 people screened for LTBI. An additional 14 people had LTBI, however, and recent transmission could be neither established nor ruled out, due to the absence of baseline test results. The Japanese study reported that, of 651 contacts meeting the WHO criteria for investigation, 25 (3.8%) had a positive IGRA result, but data on conversions were not available. Neither study identified any cases of active TB resulting from onboard transmission.

Difficulties inherent in TB epidemiology, particularly in distinguishing recent from earlier infection, complicate the interpretation of findings from contact investigations in general. Dealing with the diverse and likely geographically dispersed contacts in airline exposures presents additional challenges. Data on TST or IGRA conversions, indicating recent infection, would help to better quantify the risk of transmission following exposure on an airplane, but conducting repeat tests among passengers (who may subsequently leave the investigating country) would be logistically difficult. However, the low prevalence of positivity reported in these and other studies suggests that the risk of transmission may be low, suggesting that other TB control interventions might be prioritised over exposed air passenger screening. However, even in the absence of solid evidence of the benefit of screening air passenger contacts of active TB, and especially in situations which appear to pose a particularly high-risk, a precautionary approach may be adopted. This was part of the rationale for investigating the apparently dramatic XDR-TB incident despite a relatively short flight duration [7].

Although screening for active TB and LTBI is generally considered worthwhile, the four studies presented in this issue illustrate that substantial uncertainty remains regarding the best ways to implement screening. Critically, screening of any population is only beneficial if a positive result leads to effective action. Therefore robust systems must be in place to enable those with a positive result to access and complete treatment. Effectiveness and cost-effectiveness of strategies targeting different populations and using different diagnostic tests need to be assessed in the context of local TB epidemiology – and should account not only for the direct benefits of identifying and treating cases, but also for the reductions in incidence achieved by preventing onward transmission. As the movement of people, including those with TB, becomes increasingly common, approaches to TB control need to become correspondingly international. Cooperation within the EU and the wider international community is essential if we are to successfully control the disease.
